# Plasma hepcidin level is elevated by water immersion‐induced central fatigue via hepatic inflammatory response in male and female rats

**DOI:** 10.14814/phy2.70468

**Published:** 2025-08-04

**Authors:** Takuro Karaushi, Toshifumi Ogawa, Hiroyori Fusagawa, Taiki Kudo, Yuito Inoue, Takashi Yamada, Nobutoshi Ichise, Tatsuya Sato, Noritsugu Tohse

**Affiliations:** ^1^ Department of Cellular Physiology and Signal Transduction Sapporo Medical University School of Medicine Sapporo Japan; ^2^ Department of Cardiovascular, Renal and Metabolic Medicine Sapporo Medical University School of Medicine Sapporo Japan; ^3^ Department of Orthopedic Surgery Sapporo Medical University School of Medicine Sapporo Japan; ^4^ Graduate School of Health Sciences Sapporo Medical University Sapporo Japan; ^5^ Department of Health and Nutrition, Faculty of Human Sciences Hokkaido Bunkyo University Eniwa Japan

**Keywords:** fatigue, hepcidin, inflammation, iron metabolism, water immersion

## Abstract

Fatigue is a subjective phenomenon caused by physical or mental overexertion; however, its objective biomarkers specific to the types of fatigue remain unclear. Here, we examined whether plasma hepcidin levels, which are regulated by inflammation or iron metabolism, are elevated by peripheral and central fatigue in male and female rats. Eight‐week‐old Wistar rats were divided into three groups: peripheral fatigue, central fatigue, and sedentary control groups. Peripheral fatigue was induced by moderate‐intensity aerobic treadmill running, and central fatigue was induced by keeping rats in a cage flooded with water to a 2.5 cm depth for 5 days. Although both male and female rats showed similar behavioral phenotypes in peripheral and central fatigue groups, plasma hepcidin levels after fatigue induction were significantly elevated only in the central fatigue group. While neither iron panels nor tissue non‐heme iron levels corresponded to changes in plasma hepcidin, levels of phospho‐STAT3 at Tyr(705) in the liver were significantly elevated in both sexes in the central fatigue group, suggesting the presence of hepatic inflammation that can lead to hepcidin upregulation. The collective findings indicate that elevation of plasma hepcidin level may be a promising biomarker for central fatigue, but not for peripheral fatigue, regardless of iron metabolism alteration.

## 1 INTRODUCTION

Fatigue is often referred to as a subjective state of discomfort and a desire for rest with reduced physical activity caused by physical or mental overexertion or illness. Most cases of fatigue are reversible, and methods for transient recovery from fatigue through relaxation and massage have been practiced for a long time. As a result, studies addressing the molecular mechanisms of fatigue have not garnered much attention. However, there are unsolved fatigue‐associated health problems including overtraining syndrome, a serious condition that athletes cannot recover from even after sufficient rest (Armstrong et al., 2021). In East Asia, where there is a culture that values diligence and hard work, death from the accumulation of work‐related fatigue is called “karoshi syndrome” (Al‐Madhagi, 2023), and the increasing number of fatigue‐related health problems has become a major social concern. It has been reported that health problems caused by fatigue or overwork have become a worldwide issue due to changes in social structure (Lombardo et al., 2024; Peng et al., 2023). Furthermore, in recent years, there has been an increase in individuals suffering from various diseases accompanied by fatigue including cancer (Kang et al., 2023), cardiovascular‐kidney‐metabolic diseases (Denu et al., 2025; Singh et al., 2016), inflammatory diseases (Davies et al., 2021), and long COVID‐19, which is defined as a chronic condition that occurs after SARS‐CoV‐2 infection (Davis et al., 2023). Thus, elucidation of the molecular mechanisms underlying fatigue and establishment of novel therapeutic approaches are important issues.

Although fatigue is generally regarded as a subjective phenomenon, quantitative evaluation of fatigue is necessary to address fatigue‐related health problems. The visual analogue scale (VAS) (Yeung & Wong, 2019) and VAS to evaluate fatigue severity (VAS‐F) (Lee et al., 1991), which are subjective methods for assessing fatigue, have been widely used to assess the degree or severity of fatigue. For objective methods of assessing fatigue, several neurophysiological and functional performance measures, such as twitch interpolation and electromyogram amplitude, have been developed to quantify central and peripheral aspects of fatigue in human studies (Gandevia, 2001). However, these methods are often invasive, technically demanding, and restricted to laboratory settings. In this context, there is a strong need for accessible, minimally invasive, and pathophysiologically relevant biomarkers to evaluate fatigue objectively across different settings. There are several well‐known biomarkers, including plasma, salivary, or urine cortisol levels (Jung et al., 2014), heart rate variability (Yoshikawa et al., 2025), which is an index of the autonomic nervous system, plasma or urinary 8‐hydroxydeoxyguanosine (8‐OHdG) levels (Fukuda et al., 2016), and some serum metabolites such as lactate (Wan et al., 2017). However, many of these biomarkers primarily reflect acute stress, and their relevance to different types of fatigue remains uncertain. In addition, their diurnal variation, temporal fluctuations, and inter‐individual variability may further reduce their utility as robust indicators of fatigue severity or type. On the contrary, a recent study has demonstrated that herpes virus reactivation or its systemic response is associated with fatigue as well as a depressive state (Domingues et al., 2023; Kobayashi et al., 2020), suggesting a potent link between inflammation and fatigue. Therefore, inflammation‐related biomarkers may be a promising candidate for a novel objective indicator of central fatigue.

It has been reported that an excess or deficiency of iron, a trace metal that is essential for various cellular functions, is associated with the fatigue phenotype (Świątczak et al., 2024; Yokoi & Konomi, 2017; Zuo et al., 2016). Hepcidin, a peptide hormone that is mainly synthesized and secreted in the liver by iron levels and inflammatory cytokines, has the effect of shutting down the transfer of iron between cells by negatively regulating the iron transfer protein ferroportin1 (FPN1). During inflammation, hepcidin in the blood is increased and inhibits iron transfer, acting as an antimicrobial factor to protect the body from pathogens that require iron for their growth. In contrast, it is known that excess hepcidin is associated with various pathological conditions such as anemia, chronic kidney disease, heart failure, and some neurodegenerative diseases (Nemeth & Ganz, 2023) as well as the aging‐associated phenotype (Mezzanotte et al., 2022; Sato et al., 2022). These conditions frequently accompany chronic low‐grade inflammation and are often characterized by central fatigue, which is difficult to specifically detect using conventional markers. It is possible that a hepcidin‐fatigue‐inflammation‐iron axis is closely related to the molecular mechanism of stress‐induced central fatigue, but its details remain unclear.

Therefore, we hypothesized that hepcidin, a key regulator of iron metabolism and inflammation, may play a distinct role in differentiating between peripheral fatigue induced by aerobic exercise and stress‐induced central fatigue. To test this hypothesis, we assessed the associations of plasma hepcidin levels with systemic iron metabolism and hepatic inflammation using different fatigue rat models in the present study. Peripheral fatigue was induced by forced treadmill running under moderate‐intensity aerobic conditions, as high‐intensity exercise can elicit both peripheral and central fatigue components (Enoka & Duchateau, 2016). In contrast, central fatigue was induced by housing rats in an immersion cage designed to generate psychological stress. The experiments were conducted using both male and female rats, given the potential for sex differences in stress‐induced responses (Cairns et al., 2024) and hepcidin‐associated signaling (Kong et al., 2014).

## 2 MATERIALS AND METHODS

### 2.1 Ethical approval

Experimental protocols using animals were reviewed and approved by the Sapporo Medical University Animal Experimentation Ethics Committee (No. 23‐073), Sapporo, Japan. Animal care was carried out in strict accordance with the Guide for the Care and Use of Laboratory Animals published by the National Research Council of the National Academies, USA (2011).

### 2.2 Fatigue induction

**FIGURE 1:**
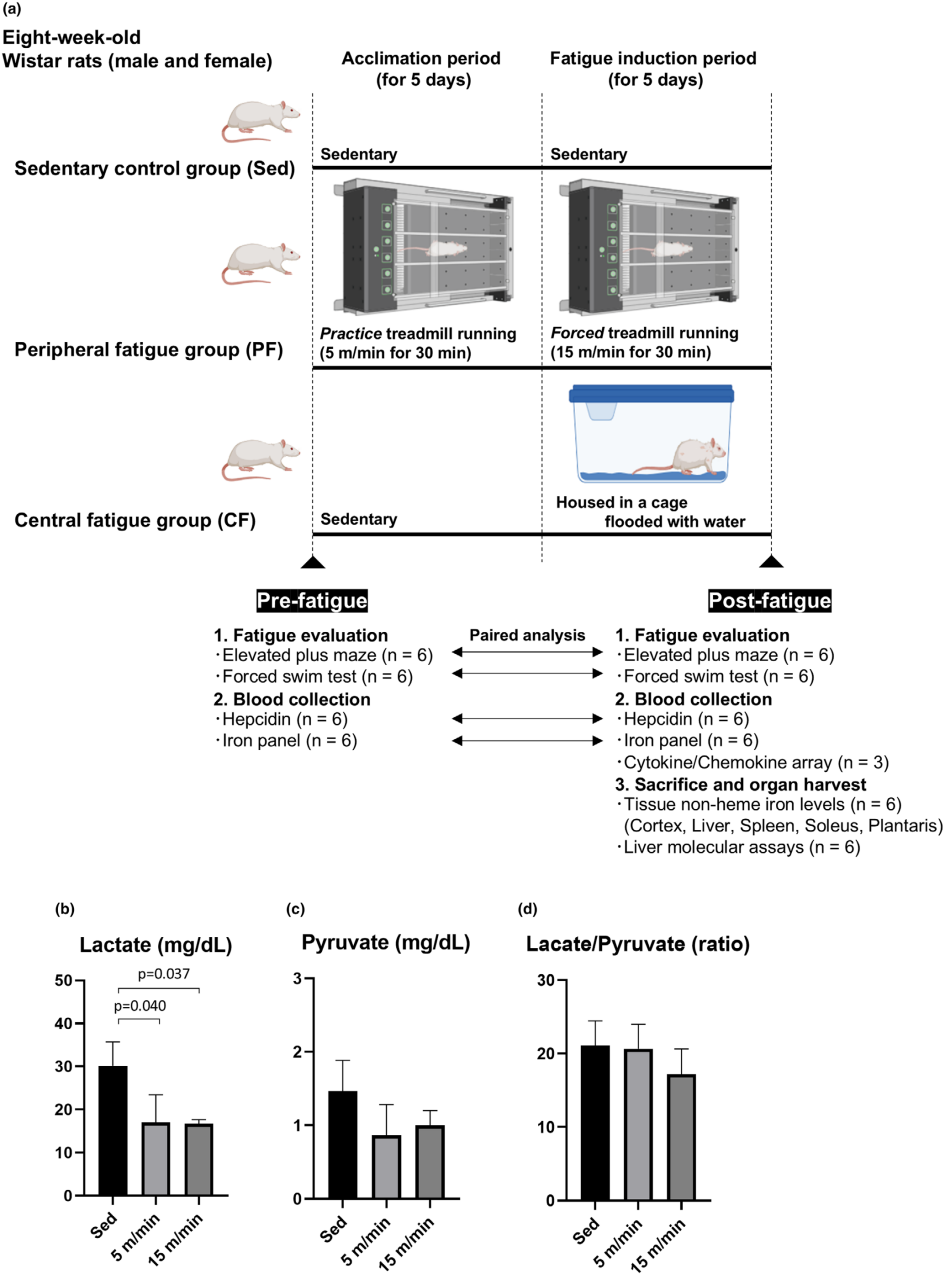
Experimental protocol in the present study. Panel a: The schematic protocol in the present study is shown. Male and female eight‐week‐old Wistar rats were divided into three groups: a sedentary control group (Sed), a peripheral fatigue group (PF), and a central fatigue group (CF). Fatigue was assessed by the elevated plus maze test and forced swim test, and levels of plasma hepcidin and iron panel were measured before (Pre‐) and after (Post‐) fatigue induction. Plasma chemokine/cytokine array, tissue non‐heme iron levels, and hepatic inflammation‐associated molecules were evaluated after fatigue induction. Although cytokine/chemokine arrays were measured with three biological replicates per group, each well of the Quantibody array contained four technical replicate spots per antibody, enabling quadruplicate measurements from a single pipetting step. Panels b–d: To determine whether exercise‐induced fatigue was aerobic or anaerobic, male Wistar rats were subjected to one of three conditions: sedentary (Sed) or practice treadmill running at 5 m/min or forced treadmill running at 15 m/min for 30 min/day for five consecutive days. Immediately after the final session, blood levels of lactate (panel b) and pyruvate (panel c) were measured, and the lactate‐to‐pyruvate ratio was calculated (panel d) (*n* = 3 per group). Statistical significance was assessed by one‐way ANOVA followed by Tukey's honestly significant difference test for post hoc comparisons.

Eight‐week‐old male and female Wistar rats (RRID:RGD_13508588) were given standard laboratory rodent chow CRF‐1 (Oriental Yeast Co., Ltd., Tokyo, Japan) and water ad libitum, and housed in an environmentally controlled room (24°C ± 2°C) with a 12‐h light–dark cycle. All rats were housed individually to eliminate potential confounding effects of social interaction or competition. The rats were randomly allocated to the following three groups: sedentary control group, peripheral fatigue group, and central fatigue group. A schematic protocol is shown in Figure 1a. Rats in the sedentary group were only moved in and out of their cages for fatigue phenotype testing, blood sampling, and weighing. Rats in the peripheral fatigue group were subjected to stress by forced treadmill exercise for 5 days at a speed of 15 m/min for 30 min/day after 5 days of acclimatization running at a speed of 5 m/min for 30 min, as previously reported with minor modifications (Fang et al., 2013; Hou et al., 2019). Rats in the central fatigue group were subjected to stress by keeping the rats in a cage flooded with water to a depth of 2.5 cm for 5 days followed by 5 days of acclimatization, as previously reported with minor modifications (Mori et al., 2021; Ogawa et al., 2012; Yasui et al., 2014; Yasui et al., 2019). A previous study reported that such a water immersion stress protocol did not significantly alter rectal temperature (Ogawa et al., 2012). The protocol was designed with a humane endpoint to euthanize the animals under anesthesia if significant weight loss (20% or more) or huddling and immobility were observed during the fatigue induction period.

### 2.3 Fatigue phenotype assessment

Before and after fatigue induction, rats were subjected to the elevated plus maze test and the forced swim test to confirm the types and degree of stress. The elevated plus maze was used primarily to assess anxiety‐like behavior, while the forced swim test was employed primarily to assess depressive‐like behavior (Erdem et al., 2024; Kraeuter et al., 2019). The elevated plus maze was made of black‐colored plastic, was elevated 50 cm from the floor, and consisted of two opposite open arms (5.0 × 30 cm). Closed arms were enclosed by 45‐cm high walls. The time spent in the open arm and the time spent in the closed arm were measured. In the forced swim test, a weight equivalent to about 10% of the body weight of a rat was attached to its tail using a clip. The rat was then made to swim in water, and the time until the rat stopped moving for 10 s was measured.

### 2.4 Sample collection

For plasma preparation, whole blood was taken from the tail vein and collected in a heparin‐coated tube (Microvette 500 Lithium heparin gel, #20.1346.100, Sarstedt, Nümbrecht, Germany). Following centrifugation at 10,000*g* for 5 min, the supernatant was collected and stored at −80°C until the assays. For organ harvest, rats were anesthetized with 2%–3% isoflurane inhalation, and after confirming that they were sufficiently anesthetized by not showing a reflex even when a strong painful stimulus was applied to the animal's hindlimb with forceps, they were euthanized by opening their chest and removing their heart. Then, tissues including the cerebral cortex, liver, spleen, soleus muscle, and plantaris muscle were excised and rinsed in phosphate buffered saline to remove excess blood. All samples were collected during the daytime in the afternoon. In the peripheral fatigue group, sampling was performed immediately after the final exercise session, while in the sedentary and water immersion groups, samples were obtained at the corresponding time point without exercise. Tissue samples were frozen by liquid nitrogen and stored at −80°C until the assays.

### 2.5 Blood lactate and pyruvate measurement

For the measurement of blood lactate and pyruvate concentrations, 500 μL of whole blood was mixed with 500 μL of ice‐cold 1.0 N perchloric acid and gently vortexed. After deproteinization, the samples were left to stand for 15 minutes and centrifuged at 1000*g* at 4°C. The resulting supernatant was separated, frozen, and sent to BML Inc. (Tokyo, Japan) for enzymatic analysis.

### 2.6 Plasma hepcidin measurement

Plasma hepcidin concentration was determined using a Rat Hepcidin ELISA kit (#CSB‐EL010124RA, Cusabio, Wuhan, China) according to the manufacturer's instructions.

### 2.7 Evaluation of plasma iron panels

Plasma iron concentration and transferrin saturation were assessed by using a ferrozine‐based iron/TIBC reagent kit (#I7504, Pointe Scientific, Canton, MI, USA) according to the manufacturer's instructions.

### 2.8 Immunoblots

Liver tissue lysate was homogenized in ice‐cold lysis buffer (#C3228, CelLytic™ MT Cell Lysis Reagent, Sigma‐Aldrich, St. Louis, MO, USA) supplemented with protease inhibitor (#786‐437, G‐Biosciences, St. Louis, MO, USA) and phosphatase inhibitor cocktail (#07574‐61, Nacalai Tesque Inc., Kyoto, Japan). The homogenate was centrifuged at 12,000*g* for 15 min at 4°C to obtain the supernatant. The protein concentration was determined using a BCA Protein Quantification Kit (#T9300A, TaKaRa Bio, Shiga, Japan). Equal amounts of protein were loaded using NuPAGE Novex 4%–12% Bis‐Tris gels (Thermo Fisher Scientific, Waltham, MA, USA) and transferred to nitrocellulose membranes. Total proteins on the membrane were visualized by 0.1% (w/v) of Ponceau S in 5% acetic acid. After blocking with TBS containing 0.005% Tween 20 and 5% milk, the membranes were incubated overnight with primary antibodies against total Stat3 (#9139, Cell Signaling, RRID:AB_331757, 1:1000 dilution), phospho‐Stat3 (Tyr705) (#9145, Cell Signaling, RRID:AB_2491009, 1:1000 dilution), and α‐Tubulin (#ab4074, Abcam, RRID:AB_2288001, 1:10000 dilution). Horseradish peroxidase‐conjugated secondary antibodies against mouse (Cytiva, Cat# NA931‐100UL, RRID:AB_772210) and rabbit (Cytiva, Cat# NA934‐100UL, RRID:AB_772206) IgG were used at dilutions of 1:4000 and 1:5000, respectively. Immunoblotted proteins were developed using an Immobilon Western detection kit (#WBKLS0100, MilliporeSigma, Billerica, MA, USA). Images were taken and processed using a ChemiDoc XRS + System with Image Lab software (RRID:SCR_014210, Bio‐Rad). Levels of the intensities of blots were normalized by total proteins detected by Ponceau S staining or internal control α‐Tubulin using Image J software (RRID:SCR_003070, National Institutes of Health, USA).

### 2.9 Measurement of hepatic interleukin‐6 levels

Liver lysate was prepared as described above and adjusted to 1.0 μg/μL. Interleukin (IL)‐6 in the liver lysate was measured using a rat IL‐6 ELISA kit (#EUR‐IL6‐CL, RayBiotech, Inc., Norcross, GA, USA) according to the manufacturer's instructions.

### 2.10 Cytokines/chemokines array

A Quantibody Rat Inflammation Array 1 (#QAR‐INF‐1‐4, RayBiotech) was used to simultaneously measure blood levels of 10 inflammatory cytokines or chemokines, including IL‐1α, IL‐1β, IL‐2, IL‐4, IL‐6, IL‐10, IL‐13, monocyte chemoattractant protein‐1 (MCP‐1), interferon‐γ (IFN‐γ), and tumor necrosis factor‐α (TNF‐α), according to the manufacturer's instructions. In brief, the glass chip was blocked by incubating it with sample diluent for 30 min. Then, standards or plasma samples were added and incubated at room temperature for 1 h. After a washing step, a detection antibody was added and incubated at room temperature for another 1 h, followed by additional washing. Subsequently, Cy3‐conjugated streptavidin was added and incubated for 1 h. Fluorescent signals were detected using a laser scanner (Axon GenePix; Molecular Devices, Sunnyvale, CA, USA) with 532‐nm excitation, and data were analyzed using RayBio Q Analyzer software (RayBiotech, Inc., Norcross, GA, USA). After background correction and normalization to positive controls, the signal intensities of antigen‐specific antibody spots were compared between groups to determine relative differences in cytokine or chemokine levels.

### 2.11 Tissue non‐heme iron levels

Tissue non‐heme iron contents were evaluated as previously described with modification (Sato et al., 2022). Briefly, frozen tissues were homogenized in ice‐cold lysis buffer (#C3228, Sigma‐Aldrich) supplemented with protease inhibitor (#786‐437, G‐Biosciences). After the lysate had been centrifuged at 3000*g* for 10 min at 4°C, the supernatant was collected. The lysate was mixed with protein precipitation solution (1:1 1 N HCl and 10% trichloroacetic acid [#T6399, Sigma‐Aldrich]) and heated at 95°C for 1 h to release protein‐binding iron. The precipitation was removed by centrifugation at 16,000*g* for 10 min at 4°C, and the supernatant was mixed with an equal volume of chromogenic solution (0.5 mM ferrozine [#160601‐1G, Sigma‐Aldrich], 1.5 M sodium acetate [#127‐09‐3, Sigma‐Aldrich], and 0.1% [v/v] thioglycolic acid [#T3758, Sigma‐Aldrich]). The absorbance was measured at 562 nm by a plate reader. The level of tissue non‐heme iron was normalized by protein concentration in the lysate determined by using a BCA Protein Quantification Kit (#T9300A, TaKaRa Bio).

### 2.12 Statistical analysis

Data are presented as means ± standard deviation (SD) for numerical data for which a normal distribution can be assumed or medians [interquartile range] for data showing skewed distributions. For paired analysis, differences in normally distributed values were analyzed by a paired *t*‐test, and differences in values with a skewed distribution were analyzed by the Wilcoxon signed‐rank test. Comparison between two independent groups was made by using an unpaired *t*‐test. When more than two groups were compared, two‐way ANOVA followed by Tukey's honestly significant difference (HSD) test was used to analyze differences between groups. All statistical analyses were performed using Graphpad prism 9.5 (RRID:SCR_002798) and differences were considered statistically significant at *p* < 0.05. Exact *p*‐values are indicated for all comparisons with *p* < 0.05.

## 3 RESULTS

### 3.1 Characteristics and behavioral phenotypes in the peripheral and central fatigue model rats

To confirm that the treadmill exercise protocol used to induce peripheral fatigue elicited aerobic metabolism, we preliminarily measured blood levels of lactate and pyruvate and calculated the lactate‐to‐pyruvate ratio after both the practice phase (5 m/min) and the forced exercise phase (15 m/min) using male rats. In both conditions, lactate levels were significantly decreased compared to sedentary controls, while pyruvate levels and the lactate‐to‐pyruvate ratio remained unchanged (Figure 1b–d). Since pyruvate is a key substrate for mitochondrial oxidative metabolism and the lactate‐to‐pyruvate ratio indirectly reflects mitochondrial respiratory function, these results support that the present exercise protocol predominantly elicited aerobic metabolism (Potolitsyna et al., 2023).

In the experimental models used in the present study, changes in body weight and food intake after fatigue induction are shown in Table 1. Male rats had a higher body weight and greater food intake than those of female rats, but there were no significant differences in body weight and food intake among the sedentary control, peripheral fatigue, and central fatigue groups.

**TABLE 1:** Information on body weight and food intake under the condition of fatigue induction in all experimental groups.

	Body weight (g)				Food intake (g/day)	
	Pre‐fatigue		Post‐fatigue		Post‐fatigue	
	Male	Female	Male	Female	Male	Female
Sedentary control group (*n* = 6)	212.7 ± 24.6	144.8 ± 18.8^a^	256.1 ± 15.6	173.5 ± 12.4^d^	20.4 ± 8.2	11.5 ± 1.9
Peripheral fatigue group (*n* = 6)	196.5 ± 19.1	155.3 ± 26.2^b^	268.1 ± 19.5	173.8 ± 7.2^e^	26.5 ± 17.5	14.8 ± 12.7
Central fatigue group (*n* = 6)	191.8 ± 24.4	137.3 ± 19.4^c^	231.7 ± 16.2	166.6 ± 11.0^f^	31.3 ± 7.0	18.8 ± 5.0

*Note*: Values are presented as mean ± SD. Two‐way ANOVA followed by Tukey's honestly significant difference test was used to assess group differences. Superscript letters (a–f) indicate statistically significant differences between groups as follows: a: vs. Pre‐Fatigue male Sedentary Group (*p* < 0.001). b: vs. Pre‐Fatigue male Peripheral Fatigue Group (*p* = 0.035). c: vs. Pre‐Fatigue male Central Fatigue Group (*p* = 0.003). d: vs. Post‐Fatigue male Sedentary Group (*p* < 0.001). e: vs. Post‐Fatigue male Peripheral Fatigue Group (*p* < 0.001). f: vs. Post‐Fatigue male Central Fatigue Group (*p* < 0.001). A significant main effect of sex was observed (*p* < 0.001) in food intake, but post hoc comparisons between subgroups did not reach statistical significance.

**FIGURE 2:**
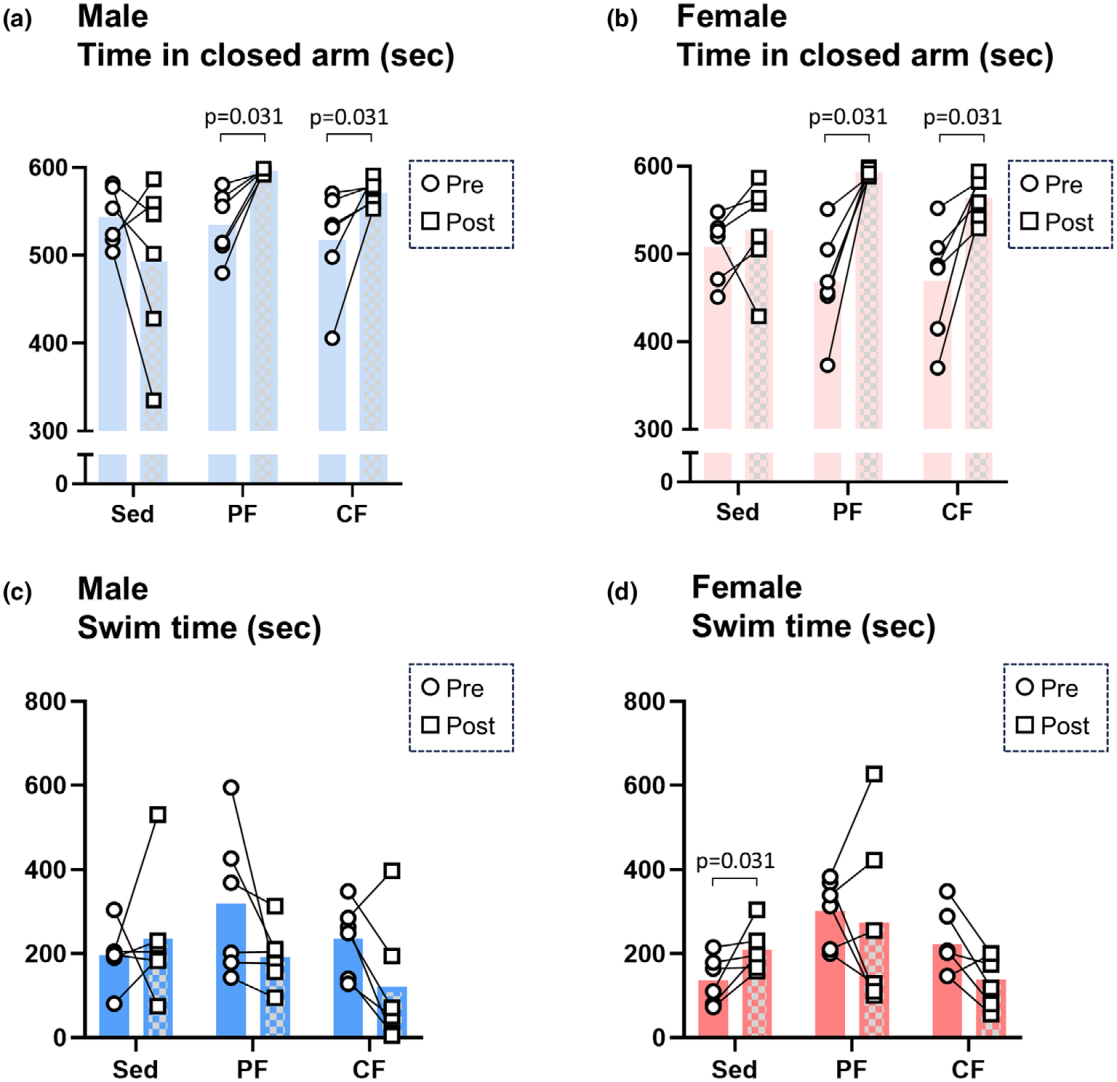
Behavioral analyses in the peripheral and central fatigue groups. Time spent in the closed arm in male rats (panel a) and female rats (panel b) before (Pre‐) and after (Post‐) fatigue induction was evaluated (*n* = 6). Swim time in male rats (panel c) and female rats (panel d) before (Pre‐) and after (Post‐) fatigue induction was evaluated (*n* = 6). Significant differences of normally distributed values were analyzed by a paired *t*‐test, and those of values with a skewed distribution were analyzed by the Wilcoxon signed‐rank test. CF, central fatigue group; PF, peripheral fatigue group; Sed, sedentary control group.

To assess fatigue‐related behavioral phenotypes, we carried out the elevated plus maze test and forced swim test for each model. The elevated plus maze test showed that the time spent in the closed arm, which reflects anxiety‐like behavior, significantly increased after fatigue induction in both the peripheral fatigue and central fatigue groups regardless of sex (Figure 2a,b). On the contrary, the forced swim test showed that the swim time, which reflects depressive‐like behavior, did not change in either the peripheral fatigue group or the central fatigue group regardless of sex (Figure 2c,d). These results indicate that the models used in the present study showed an increase in anxiety‐like behavior in both the peripheral fatigue group and the central fatigue group, and these characteristics were observed in both male and female rats.

### 3.2 Plasma hepcidin levels are elevated independently of iron panels after central fatigue induction in both male and female rats

**FIGURE 3:**
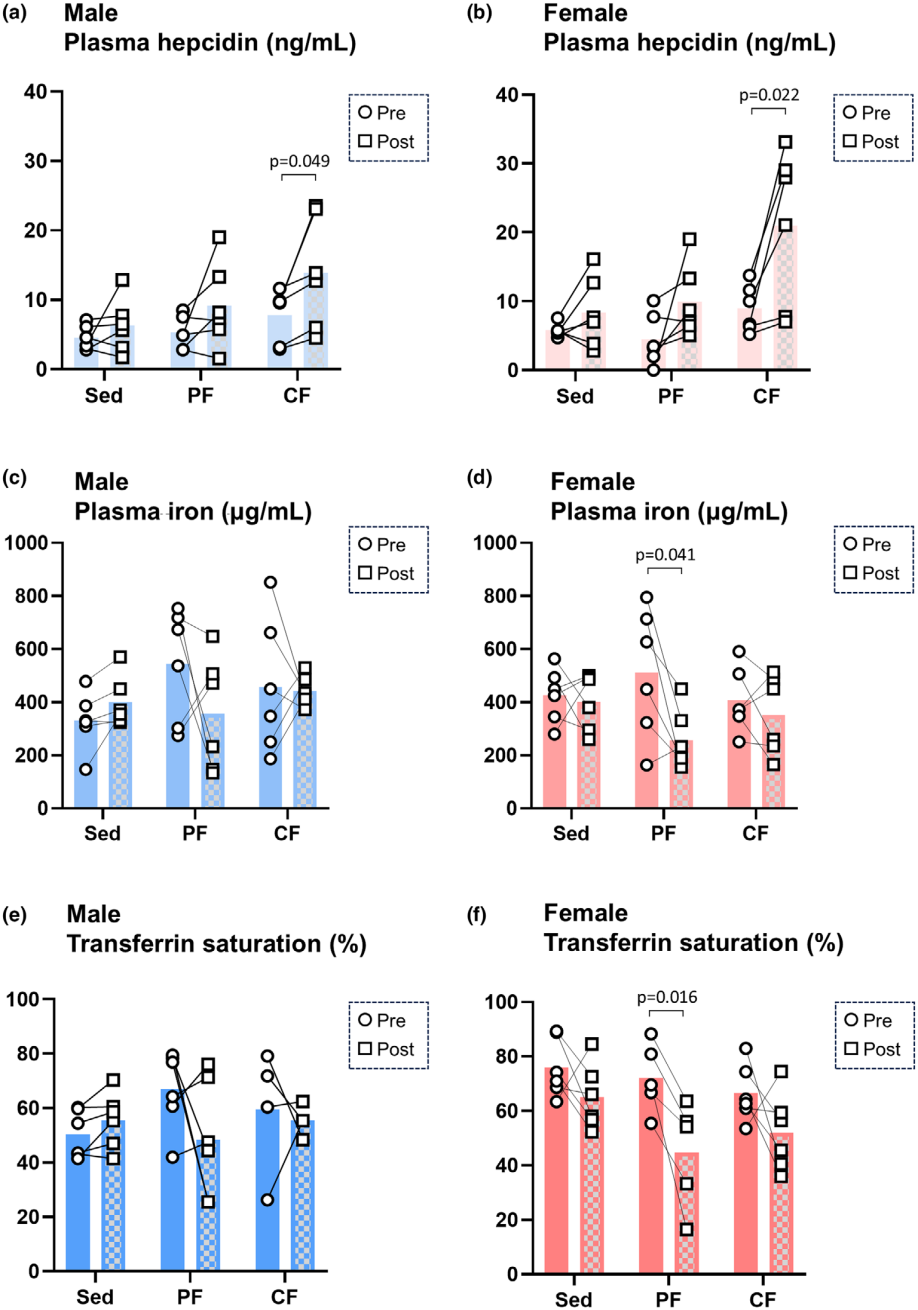
Levels of plasma hepcidin were increased after fatigue induction in the central fatigue group irrespective of changes in iron panels. Plasma hepcidin levels in male rats (panel a) and female rats (panel b) before (Pre‐) and after (Post‐) fatigue induction were evaluated (*n* = 6). Plasma iron levels in male rats (panel c) and female rats (panel d) before (Pre‐) and after (Post‐) fatigue induction were evaluated (*n* = 6). Transferrin saturation in male rats (panel e) and female rats (panel f) before (Pre‐) and after (Post‐) fatigue induction was evaluated (*n* = 6). Significant differences of normally distributed values were analyzed by a paired *t*‐test, and those of values with a skewed distribution were analyzed by the Wilcoxon signed‐rank test. CF, central fatigue group; PF, peripheral fatigue group; Sed, sedentary control group.

Plasma hepcidin concentrations before and after fatigue induction were evaluated for each model. As in the sedentary control group, both male and female rats in the peripheral fatigue group showed no significant changes in plasma hepcidin levels after fatigue induction (Figure 3a,b). However, in the central fatigue group, there were significant increases in plasma hepcidin concentrations in both male rats (1.78‐fold increase) and female rats (2.34‐fold increase) after fatigue induction (Figure 3a,b).

Since iron panels, which consist of circulating iron concentration and transferrin saturation, can both regulate and be regulated by plasma hepcidin levels, we then evaluated the iron concentration and transferrin saturation in the plasma of each model. In contrast to the changes in plasma hepcidin levels, there were no changes in plasma iron levels and transferrin saturation after fatigue induction in the central fatigue group (Figure 3c–f), whereas plasma iron concentration and transferrin saturation showed significant decreases in female rats in the peripheral fatigue group after fatigue induction (Figure 3d,f). These findings indicate that increases in circulating hepcidin levels induced by central fatigue induction were at least independent of changes in iron panels.

### 3.3 Inflammatory signaling in the liver is enhanced with increased local IL‐6 levels by central fatigue induction in both male and female rats

**FIGURE 4:**
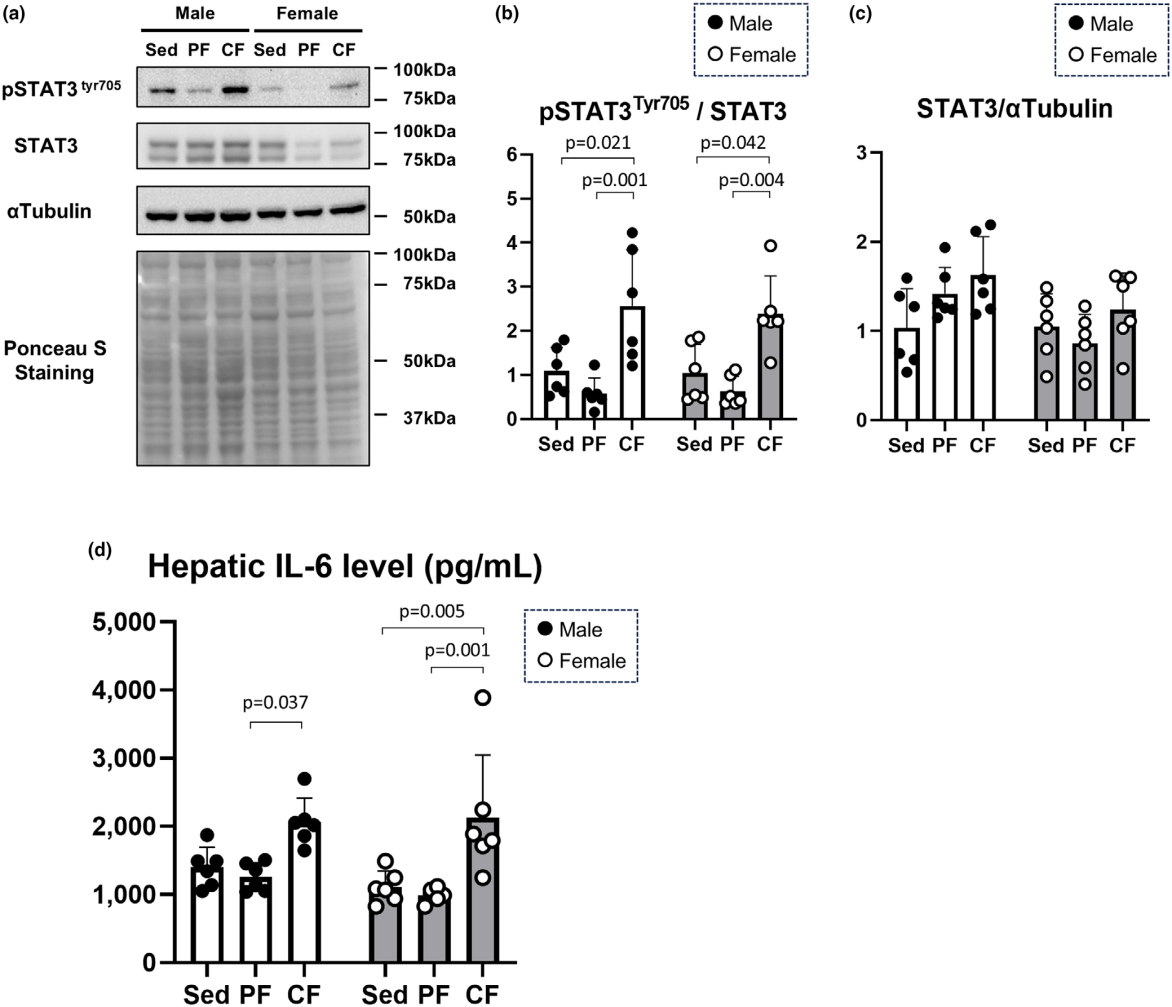
Hepatic inflammatory signaling was enhanced in the central fatigue group. Representative immunoblots of pSTAT3^tyr705^, STAT3, and α‐tubulin in liver lysates in each fatigue group (Panel a) and their densitometries (Panels b and c) (*n* = 6). Levels of IL‐6 in the liver lysate in each fatigue group were evaluated (panel d) (*n* = 6). Significant differences between groups were evaluated by two‐way ANOVA followed by Tukey's honestly significant difference test. pSTAT3^tyr705^: Phosphorylated signal transducers and activator of transcription 3 at tyrosine 705. IL‐6, interleukin‐6; STAT3, signal transducers and activator of transcription 3.

Since most of the circulating hepcidin is derived from the liver and our findings showed that the secretion of hepcidin due to fatigue may be regulated by inflammation rather than iron metabolism, we finally evaluated the STAT3‐mediated pathway, which is essential for hepcidin expression due to inflammation (Nemeth & Ganz, 2023). Levels of phosphor‐STAT3 at Tyr(705) in the liver were significantly elevated in both male and female rats in the central fatigue group, while the levels tended to be rather decreased in both male and female rats in the peripheral fatigue group (Figure 4a,b). There was no significant difference in STAT3 expression levels among the groups (Figure 4a,c).

IL‐6‐mediated signaling has been known to be a major upstream modulator of STAT3 (Nemeth & Ganz, 2023). Thus, we measured IL‐6 levels in liver lysates. In male rats, hepatic IL‐6 levels were significantly higher in the central fatigue group than in the peripheral fatigue group, whereas in female mice, hepatic IL‐6 levels were significantly elevated in the central fatigue group compared with the levels in the sedentary control and peripheral fatigue groups (Figure 4d). The collective findings indicate that the increase in plasma hepcidin levels with central fatigue induction is mediated by hepatic inflammation with STAT3‐mediated signaling, at least partially through local IL‐6 levels.

### 3.4 Plasma inflammatory cytokines and chemokines exhibited various changes depending on the type of fatigue and sex

**FIGURE 5:**
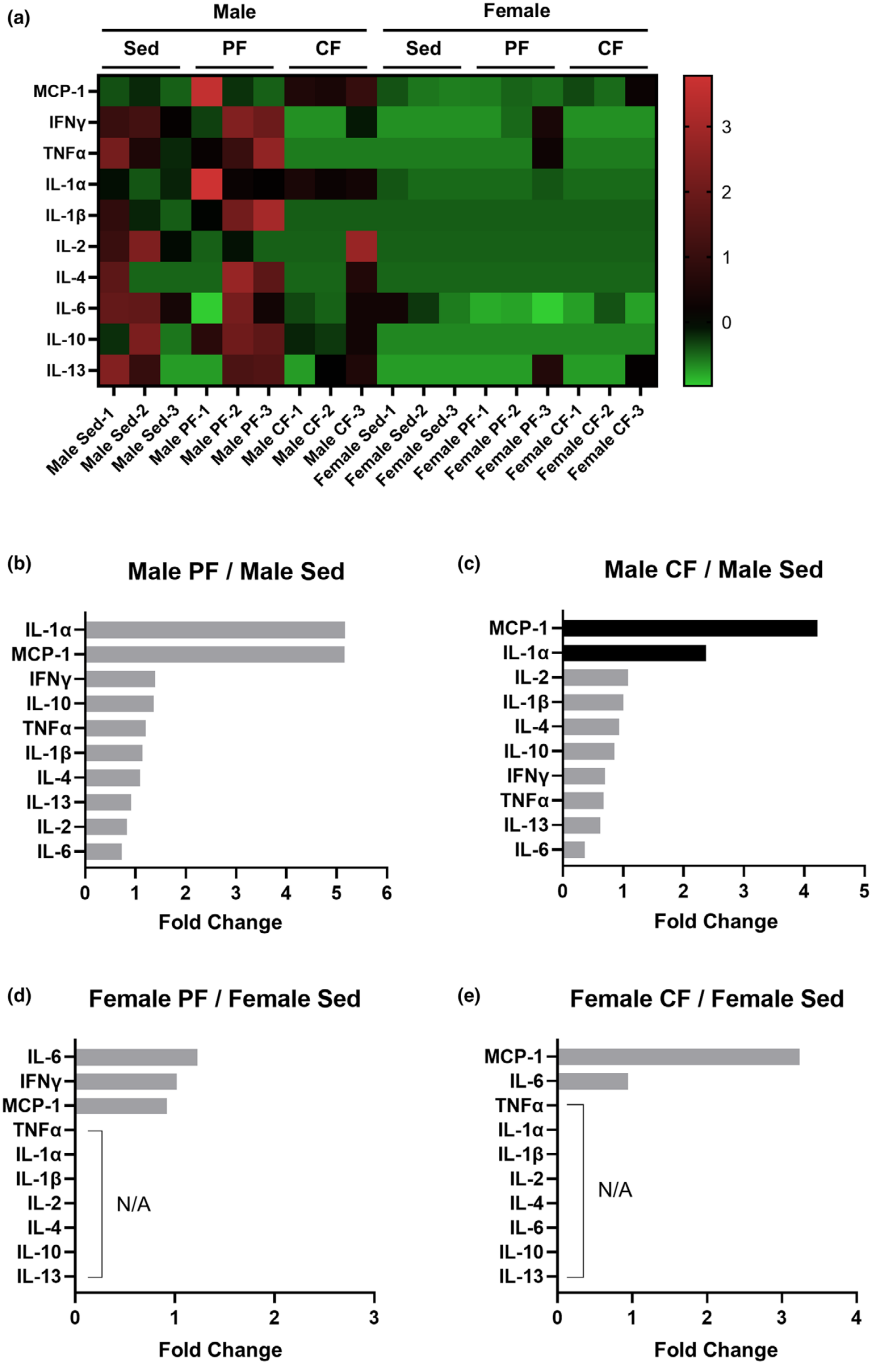
Plasma cytokine/chemokine array in the peripheral and central fatigue groups. Cytokine/chemokine measurements were obtained using the Quantibody Rat Inflammation Array 1, which includes four replicate antibody spots per cytokine per well. The values of the cytokine/chemokine array were standardized (*μ* = 0, *σ* = 1) and displayed as a heat map (panel a). The values were ranked in order of magnitude, with the fatigue group having higher values than those in the sedentary control group (panel b: Male PF vs. male Sed, panel c: Male CF vs. male Sed, panel d: Female PF vs. female Sed, panel e: Female CF vs. female Sed). The intensity of the bars reflects statistical significance: Darker bars represent comparisons with *p* < 0.05, whereas lighter bars indicate non‐significant differences (*p* ≥ 0.05) as assessed by a two‐tailed *t*‐test. CF, central fatigue; IFN, interferon; IL, interleukin; N/A, not applicable; MCP‐1, monocyte chemotactic protein‐1; PF, peripheral fatigue; Sed, sedentary control; TNF‐α, tumor necrosis factor‐α.

Next, to investigate the relationship between the increased plasma hepcidin levels and systemic inflammation in the central fatigue model, a comprehensive cytokine and chemokine array was evaluated using plasma samples collected after fatigue induction. Heatmap analysis showed that the cytokines and chemokines evaluated in the present study were markedly lower in female rats than in male rats (Figure 5a). The levels of IFN‐γ, TNF‐α, IL‐1α, IL‐1β, IL‐4, IL‐6, IL‐10, and IL‐13 were significantly lower in female rats than in male rats (Figure S1). In rank‐based analysis, the top three cytokines or chemokines that were more abundant in the peripheral fatigue group than in the sedentary control group in male rats were IL‐1α, MCP‐1, and IFN‐γ (Figure 5b), while those in the central fatigue group were MCP‐1, IL‐1α, and IL‐2 (Figure 5c). On the contrary, in female rats, IL‐6 and MCP‐1 were the cytokines or chemokines that were mostly increased by peripheral fatigue and central fatigue, respectively (Figure 5d,e). These findings suggest that there are sex differences in fatigue‐induced changes in plasma cytokines and chemokines depending on the type of fatigue. Notably, fatigue‐induced changes in MCP‐1 and IL‐1α were predominant in male rats, and fatigue‐induced changes in MCP‐1 and IL‐6 were predominant in female rats, whereas levels of chemokines or cytokines in plasma did not predict the change in plasma hepcidin levels induced by fatigue.

### 3.5 Tissue non‐heme iron levels are not affected by peripheral or central fatigue induction

**FIGURE 6:**
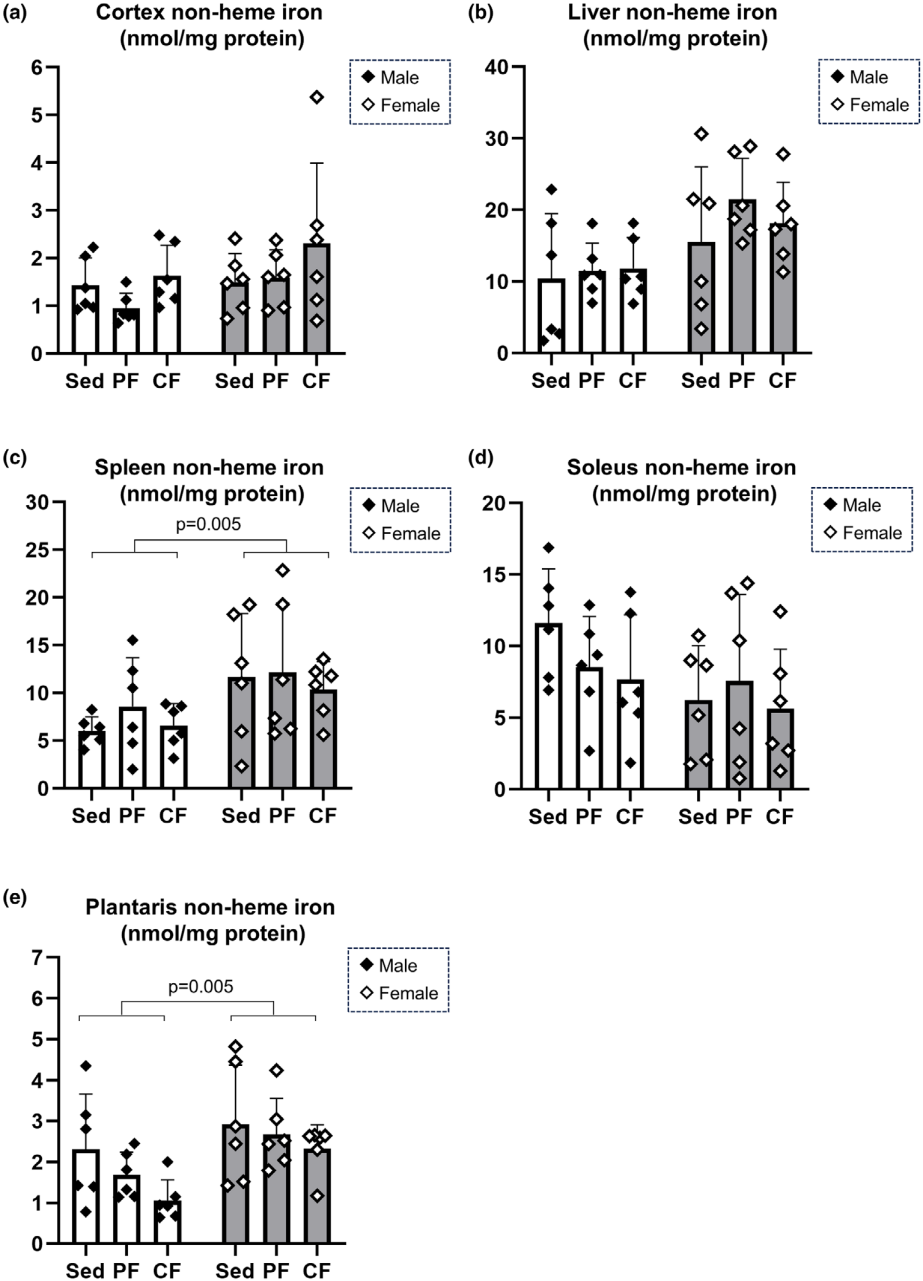
Evaluation of tissue non‐heme iron levels. Non‐heme iron levels in the cortex (panel a), liver (panel b), spleen (panel c), soleus (panel d) and plantaris (panel e) were measured (*n* = 6). Significant differences between groups were evaluated by two‐way ANOVA, and post hoc analyses were carried out by Tukey's honestly significant difference test.

Finally, since changes in circulating hepcidin levels can be associated with iron metabolism throughout the body, we examined the concentrations of non‐heme iron in total lysates of the cerebral cortex, liver, spleen, soleus muscle as a representative muscle of slow‐twitch muscles, and plantaris muscle as a representative muscle of fast‐twitch muscles (Figure 6). The concentrations of non‐heme iron in the spleen and plantaris muscle were significantly increased in female rats compared with those in male rats regardless of the type of fatigue (Figure 6c,e). Although non‐heme iron concentration in the plantaris muscle of male rats in the central fatigue group tended to decrease, the changes did not reach statistical significance (Figure 6e). There were no significant changes in non‐heme iron concentrations in the other organs in each group (Figure 6a–e). These results suggest that the increased levels of circulating hepcidin in central fatigue at least can be induced independently of systemic iron metabolism.

## 4 DISCUSSION

The present study showed that central fatigue, but not peripheral fatigue, is accompanied by an increase in plasma hepcidin levels along with hepatic Tyr 705 phosphorylation of STAT3, which reflects inflammatory signaling in the liver, regardless of sex. The findings suggest that the elevation of plasma hepcidin and hepatic inflammation are essential factors for distinguishing fatigue phenotypes, even if the fatigue phenotypes are similar. Notably, hepatic inflammation induced by central fatigue cannot be explained by plasma cytokine or chemokine levels, and our results indicate that elevated plasma hepcidin may be a novel and useful indicator for differentiating between types of fatigue.

In accordance with our results, previous studies have shown that mental overexertion and its disorders can lead to activation of hepatic inflammation with activation of the hypothalamic–pituitary–adrenal (HPA) axis (Liu, Zhang, et al., 2023). Regarding the HPA axis, stress‐induced activation of the central nervous system causes neuroendocrine reactions that activate inflammation mainly via the secretion of endogenous glucocorticoids, including corticosterone in rodents or cortisol in humans (Nandam et al., 2019). In addition, chronic sympathetic nerve activation and noradrenaline‐related signaling in the liver, regardless of activation of the HPA axis, may play an important role in hepatic inflammation in mental stress‐induced immune response (Zhou et al., 2024; Zou et al., 2024). Peripheral fatigue also can induce an increase in inflammatory cytokines due to exercise, although its degree depends on the intensity and duration of the exercise (Enoka & Duchateau, 2016; Scheffer & Latini, 2020). Given that inflammation can be an upstream response to fatigue, regardless of its type, objectively distinguishing between central and peripheral fatigue is challenging. Conventional biomarkers, such as glucocorticoids, physical indices reflecting sympathetic nerve activation, and C‐reactive protein (CRP), which is elevated in “systemic” inflammation, have inherent limitations in differentiating peripheral and central fatigue types. However, in the present study, the treadmill‐based peripheral fatigue protocol was confirmed to be aerobic and did not result in increased hepatic IL‐6 levels or hepcidin elevation. This finding strongly suggests that moderate‐intensity aerobic exercise may not sufficiently activate the HPA axis or elicit liver‐targeted inflammation. In this context, the results of the present study showed that hepcidin levels in blood preferentially increase in central fatigue, reflecting hepatic inflammation, and suggest the potential of hepcidin level as a novel biomarker for distinguishing different types of fatigue.

Our plasma cytokine/chemokine array analysis revealed distinct inflammatory profiles in the bloodstream between peripheral fatigue and central fatigue, with MCP‐1 elevation being a characteristic feature of central fatigue, particularly in male rats. MCP‐1, also known as C‐C motif chemokine ligand 2 (CCL2), is a chemokine that primarily promotes the migration of monocytes and macrophages, contributing to the formation of an inflammatory environment (Chen et al., 2023). The dramatic increase in plasma MCP‐1 observed in central fatigue in the present study may contribute to the induction of immune cell infiltration into the liver. In addition, IL‐1α, a proinflammatory cytokine that plays a crucial role in the initiation and propagation of inflammatory responses, showed a trend to increase after fatigue induction in male rats, regardless of the degree of fatigue. Indeed, an increase in IL‐1α has been reported to trigger inflammatory responses in fatigue‐related brain regions such as the hypothalamus and amygdala, which may be associated with the subjective enhancement of fatigue sensation (Roerink et al., 2017). Additionally, important findings were obtained regarding the levels of plasma IL‐6. To our surprise, blood IL‐6 levels did not show significant changes in either peripheral or central fatigue. Considering that the well‐known IL‐6 downstream signaling, particularly hepcidin production via STAT3 phosphorylation (Wrighting & Andrews, 2006), was markedly activated in the liver after central fatigue induction, it is possible that the characteristics of cytokine changes in the bloodstream and the liver differ in response to fatigue. Alternatively, since MCP‐1 has been reported to be interrelated with IL‐6 (Brasier, 2010; Niwa et al., 2016), central fatigue may accelerate IL‐6‐related signaling in tissues with increased MCP‐1 in the blood. Indeed, our findings showed that local IL‐6 levels in the liver tended to be increased by central fatigue induction. Nevertheless, the present study showed that plasma cytokines/chemokines undergo various changes due to fatigue, with differences in the degree of change between males and females. While these cytokines and chemokines may play a role in hepatic inflammation and the pathophysiology of fatigue, our findings suggest that measuring plasma hepcidin is a more promising way of distinguishing between types of fatigue.

Despite an increase in plasma hepcidin levels in central fatigue, only a trend toward a decrease was observed in the non‐heme iron content of the plantaris muscle in males, with no significant effects on plasma iron parameters or iron contents of major systemic organs. Hepcidin shuts down iron transport by downregulating the iron transport protein FPN1 via ubiquitination, thereby reducing intestinal iron absorption, restricting iron release from the liver and macrophages, and potentially lowering serum iron levels (Nemeth & Ganz, 2023). The exact reason why there were no changes in iron metabolism despite the increase in hepcidin in the central fatigue group in the present study is unknown, but one possible reason is that the fatigue induction adopted in the present study was relatively short‐term. Since iron is an essential trace metal for cellular functions, iron metabolism is physiologically regulated by multiple mechanisms at the cellular and systemic levels. Also, iron metabolism is maintained by a balance between supply and consumption, and it is influenced by factors such as iron loss through sweating from intense exercise, gastrointestinal losses, and the degree of cellular iron recycling. Indeed, the present study showed significant reductions in plasma iron levels and transferrin saturation after peripheral fatigue induction, especially in female rats, despite unchanged plasma hepcidin levels. Previous studies have shown varying findings regarding whether exercise leads to an increase in hepcidin levels (Fernández‐Lázaro et al., 2020; Larsuphrom & Latunde‐Dada, 2021; Ryan et al., 2021; Zügel et al., 2019), presumably due to factors such as the type, intensity, duration, and individual background of the exercise, which may influence the results. Furthermore, a reduction in plasma iron can negatively regulate hepatic hepcidin production. Therefore, the absence of a significant increase in hepcidin in peripheral fatigue in the present study could rationally be explained by a negative feedback mechanism driven by hepcidin‐independent plasma iron depletion. Nevertheless, while hepcidin serves as a master regulator of iron metabolism, the results of the present study showed that hepcidin not only functions as an iron‐regulatory molecule via FPN1 but can also reflect hepatic inflammatory signaling irrespective of iron metabolism.

From the perspective of interventions aimed at alleviating fatigue, there is evidence suggesting a distinct link between liver inflammation and fatigue phenotype (D'Mello & Swain, 2014; Liu, Gong, et al., 2023). Given the findings of the present study, mitigating inflammation in the liver may be a target for alleviating central fatigue. One of the most realistic ways to recover from central fatigue is exercise (Yu et al., 2024). While exercise is an established strategy for ameliorating inflammation (Bianchi et al., 2021; Scheffer & Latini, 2020), care must be taken with the intensity and type of exercise. It has recently been reported that even mild exercise, Yoga, a traditional mind–body practice that originated in ancient India, can modulate inflammation induced by obesity via regulating adipokines such as adiponectin and leptin (Shimizu et al., 2025). In fact, even mild exercise is recommended for patients with chronic diseases such as diabetes to maintain their mental health as well as improve disease management (Mohammad Rahimi et al., 2022). In addition to appropriate exercise, pharmacological inhibitors against MCP‐1 and IL‐6 have been reported to inhibit hepatic inflammation (Baeck et al., 2012; Song et al., 2010), although adverse effects by modulating the immune response should be considered (Deshmane et al., 2009; Wang et al., 2024). Future studies are needed to investigate the effects of exercise and pharmacological approaches targeting liver inflammation on fatigue management, taking into account different fatigue types and their underlying pathophysiological mechanisms.

## 5 LIMITATIONS

The present study has several limitations. First, the objective evaluation of fatigue remains inherently difficult, as fatigue is a subjective and multifaceted phenomenon involving both physical and psychological components. Although fatigue was assessed using two selected behavioral tests, including the elevated plus maze and forced swim tests in the present study, these approaches may not fully capture the complexity and spectrum of fatigue states. Moreover, only a single level of intensity and duration was applied for each fatigue model, which may limit generalizability. To more comprehensively understand the relationship between fatigue and hepcidin, future studies should incorporate diverse assessment methods and experimental conditions, including varying degrees and durations of fatigue. Second, tissue iron levels were assessed only on the basis of non‐heme iron in whole cell lysates. Since iron distribution varies depending on physiological conditions (Sato et al., 2022), evaluation of iron content in different cellular compartments, such as the cytosol and mitochondria, may be essential in various fatigue models. In addition, heme iron, as a functionally significant form of intracellular iron as well as non‐heme iron, may play a critical role in the fatigue‐inflammation‐hepcidin‐iron axis, although heme iron levels could not be assessed in the present study. In fact, it has been reported that a deficiency of heme iron can induce muscle atrophy (Akabane et al., 2024). The possibility that heme iron, rather than non‐heme iron, plays an important role in the mechanism of muscle atrophy caused by stress or central fatigue and inflammation‐iron‐muscle atrophy is an important research topic in the future. Third, the present study was conducted using rats, and the applicability of the results to chronic fatigue or stress‐related disorders in humans remains uncertain. The regulatory mechanisms of hepcidin also may differ between species, which must be carefully considered when extrapolating findings from rats to humans. In addition, sex differences observed in the present study should be interpreted with caution because the estrous cycle in rats differs from the menstrual cycle in humans. Moreover, aging is known to influence iron metabolism and hepcidin regulation (den Elzen et al., 2013; Sato et al., 2022), which may affect the physiological response to fatigue. Based on the findings of this study, further research in humans is warranted to investigate the roles of fatigue, hepcidin, and hepatic inflammatory signaling in relation to the severity of central fatigue, with careful consideration of both sex and age‐related factors.

## 6 CONCLUSIONS

Plasma hepcidin levels were significantly increased by 5‐day water immersion‐induced central fatigue in both male and female rats. Such change was associated with enhanced hepatic inflammatory signaling via STAT3 phosphorylation irrespective of systemic chemokines, cytokines, or iron metabolism. The increase in plasma hepcidin levels, which reflects hepatic inflammation, may be an objective and promising blood biomarker for distinguishing central fatigue from peripheral fatigue.

## AUTHOR CONTRIBUTIONS

Conceptualization, Takuro Karaushi, Tatsuya Sato, and Noritsugu Tohse; methodology, Takuro Karaushi, Toshifumi Ogawa, Tatsuya Sato, and Noritsugu Tohse; software, Takuro Karaushi, Toshifumi Ogawa, Hiroyori Fusagawa, and Tatsuya Sato; validation, Takuro Karaushi, Toshifumi Ogawa, Tatsuya Sato, and Noritsugu Tohse; formal analysis, Takuro Karaushi, Toshifumi Ogawa, Hiroyori Fusagawa, Nobutoshi Ichise, and Tatsuya Sato; investigation, Takuro Karaushi, Toshifumi Ogawa, Hiroyori Fusagawa, Taiki Kudo, Yuito Inoue, Takashi Yamada, Nobutoshi Ichise, and Tatsuya Sato; resources, Takuro Karaushi, Toshifumi Ogawa, Tatsuya Sato, and Noritsugu Tohse; data curation, Takuro Karaushi, Toshifumi Ogawa, Hiroyori Fusagawa, Taiki Kudo, Yuito Inoue, and Tatsuya Sato; writing‐original draft preparation, Takuro Karaushi and Tatsuya Sato; writing‐review and editing, Takuro Karaushi, Toshifumi Ogawa, Tatsuya Sato, and Noritsugu Tohse; visualization, Takuro Karaushi, Toshifumi Ogawa, and Tatsuya Sato; supervision, Takashi Yamada, Nobutoshi Ichise, Tatsuya Sato, and Noritsugu Tohse; project administration, Takuro Karaushi, Tatsuya Sato, and Noritsugu Tohse. All authors have read and agreed to the published version of the manuscript.

## FUNDING INFORMATION

This research was partly supported by grants from the Japan Society for the Promotion of Science (20K18069 and 19K08522) and was supported by Sapporo Medical University Grants for Programs promoting Academic advancements.

## CONFLICT OF INTEREST STATEMENT

The authors declare no conflict of interest related to this project.

## Supporting information


Figure S1.

Figure S2.

## Data Availability

The data that support the findings of the present study are available on request from the corresponding author.
