# Supplementary figures and images for: Plasma hepcidin level is elevated by water immersion‐induced central fatigue via hepatic inflammatory response in male and female rats
Source: Physiol Rep. 2025 Aug 4;13(15):e70468. doi: 10.14814/phy2.70468 (PMC12320131; doi:10.14814/phy2.70468)

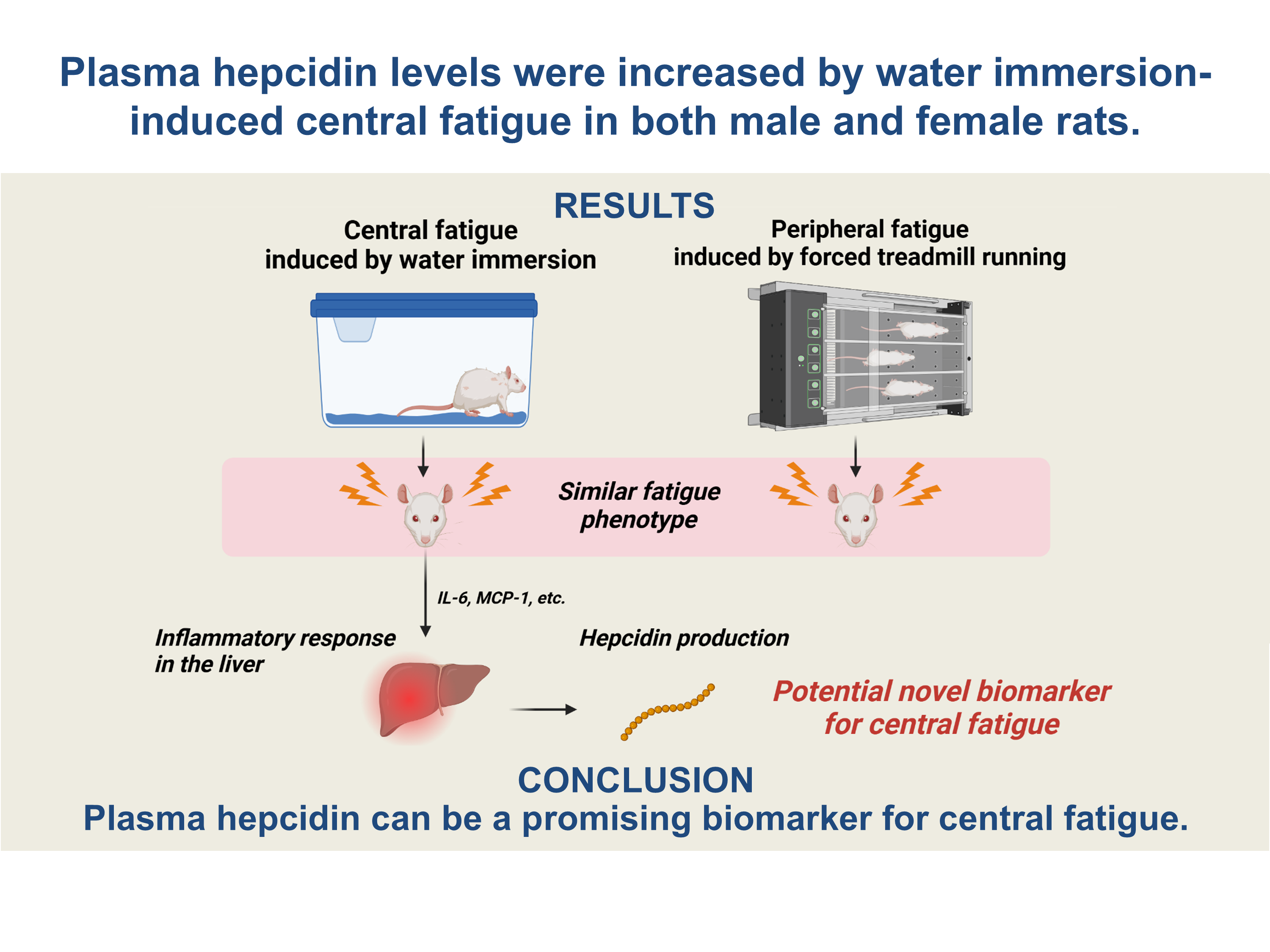

Supplement: Supplementary file 1 — Figure S1. [file PHY2-13-e70468-s001.tif]

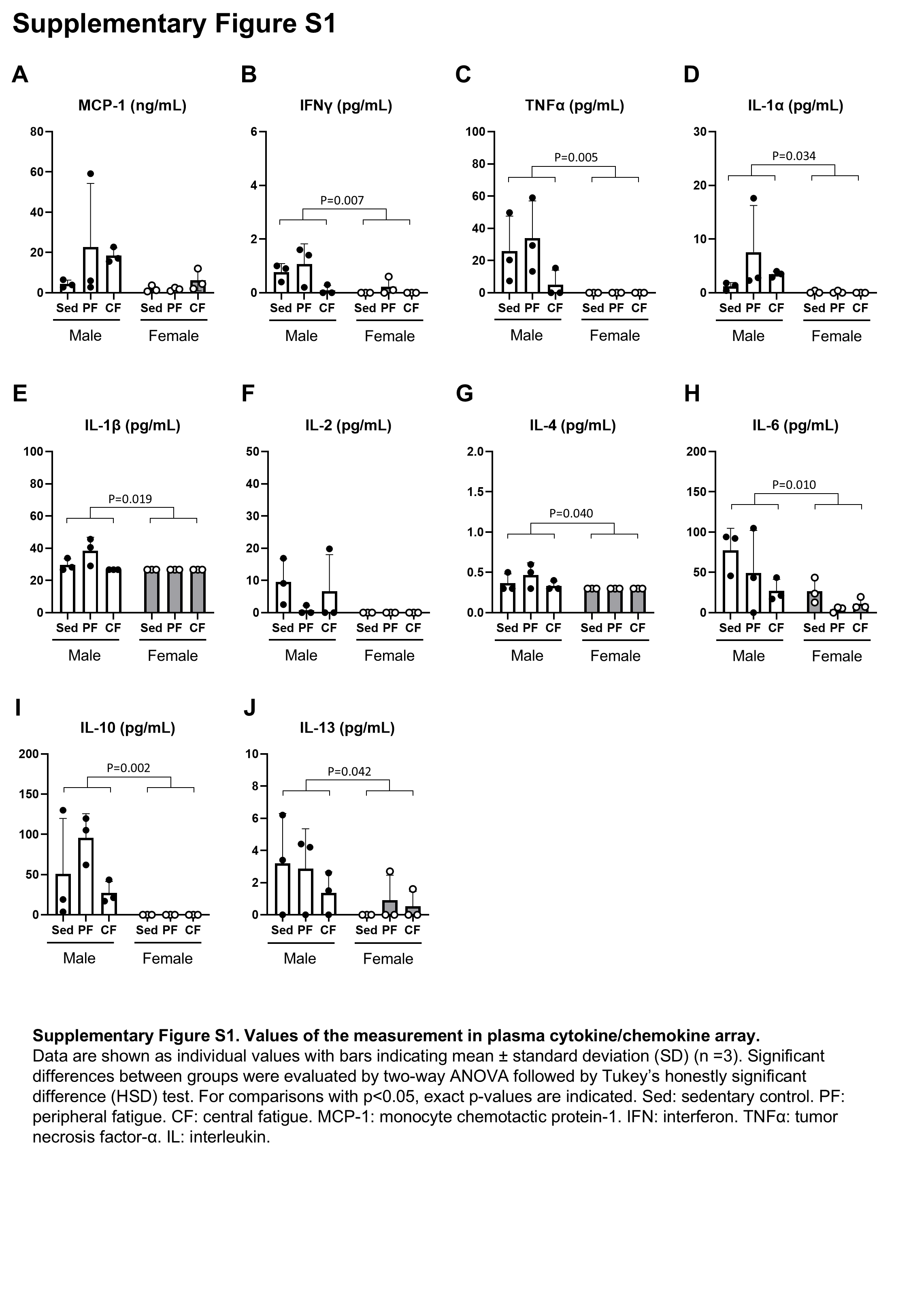

Supplement: Supplementary file 2 — Figure S2. [file PHY2-13-e70468-s002.tif]
